# P-1239. Pharmacist-Driven Beta-Lactam Therapeutic Drug Monitoring: Initial Results from In-House Pilot

**DOI:** 10.1093/ofid/ofaf695.1431

**Published:** 2026-01-11

**Authors:** Katie B Olney, Donna R Burgess, Danielle Casaus, Nicole Slain, Will Harris, Hunter Curry, David S Burgess

**Affiliations:** University of Kentucky HealthCare, Lexington, KY; UK HealthCare, Lexington, KY; University of Kentucky HealthCare, Lexington, KY; University of Kentucky HealthCare, Lexington, KY; University of Kentucky HealthCare, Lexington, KY; University of Kentucky HealthCare, Lexington, KY; University of Kentucky, Lexington, KY

## Abstract

**Background:**

Institutional implementation of beta-lactam therapeutic drug monitoring (TDM) requires aligned infrastructure, physician and pharmacist buy-in, and coordinated collaboration. At our institution, TDM is managed by pharmacists who are granted institutional authority to dose, order, and assess levels and labs as clinically indicated. Following validation of in-house assays for cefepime (CFP), meropenem (MER), and piperacillin (PIP), pharmacist-driven TDM for these three agents was piloted in the intensive care unit (ICU) and select medical wards.

**Figure:**
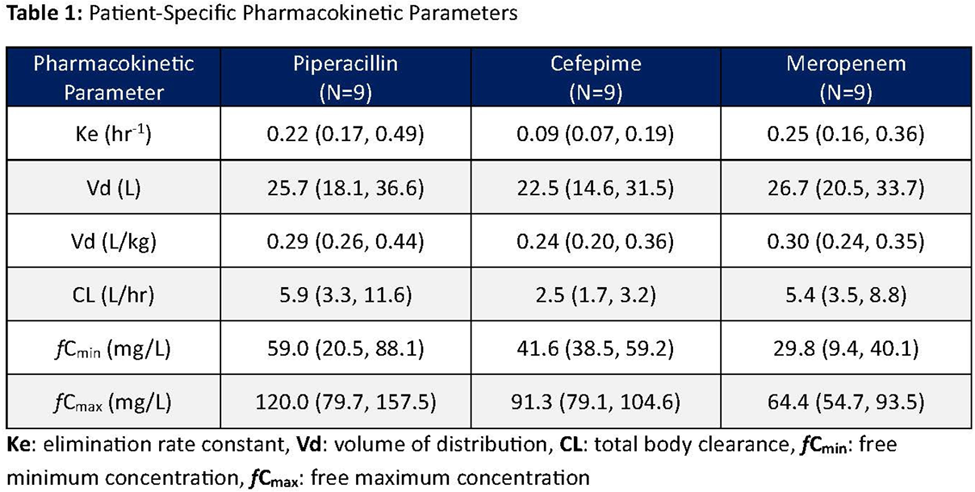

**Figure:**
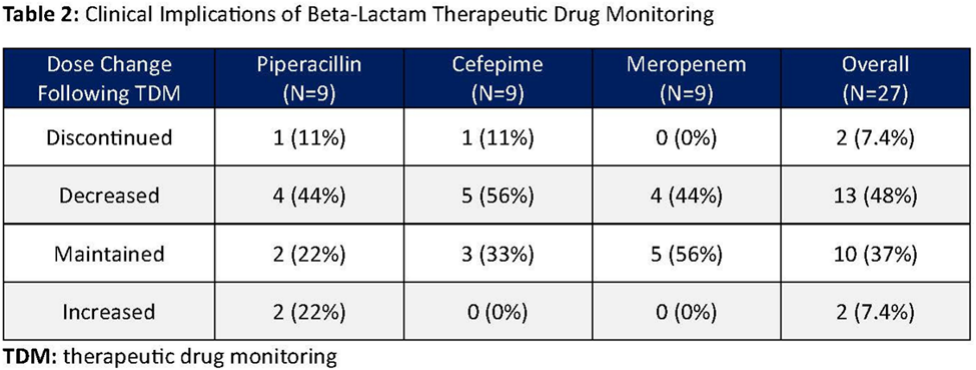

**Methods:**

This single-center, prospective study evaluated the impact of pharmacist-driven TDM in adult inpatients admitted to an ICU or medical ward at UK HealthCare receiving CFP, MER, or PIP and had two steady-state concentrations available for analysis. In-house assays provided results within 24 hours with estimation of free drug concentration based on protein binding (PIP: 30%, CFP: 20%, MER: 0%). Pharmacokinetic (PK) parameters were calculated using first-order equations, and the pharmacodynamic (PD) target was 100% *f*T >1–4×MIC. Pharmacists used individualized PK to ensure PD target attainment and all interventions were recorded.

**Results:**

Twenty-seven patients were evaluated (ICU: n=22, non-ICU: n=5). Median (IQR) age was 61 years (50, 69), weight 82.5 kg (72.8, 102), BMI 28.2 kg/m² (25.8, 30.8), and 67% were male. Each drug was administered to 9 patients. Fifteen patients had an identified organism: *P. aeruginosa* (n=10), ESBL-producing organisms (n=2), *S. marcescens* (n=2), and other (n=1); 12 received empiric therapy. PK parameters for each agent are outlined in Table 1. Across all agents, 48% (n=13) required dose reductions, 7% (n=2) had doses increases (PIP only), only 37% (n=10) were maintained, and 7% (n=2) were discontinued (Table 2).

**Conclusion:**

Pharmacist-driven beta-lactam TDM identified substantial PK variability and led to individualized dose optimization in the majority of cases. This approach improved target attainment and highlights the clinical and operational value of integrating real-time TDM into antimicrobial stewardship strategies. As institutions strive to align stewardship with precision medicine, beta-lactam TDM may no longer be a luxury, but a necessity.

**Disclosures:**

All Authors: No reported disclosures

